# Radiotherapy-associated pleomorphic dermal sarcoma 33 years after basal cell carcinoma treatment

**DOI:** 10.1016/j.jdcr.2026.05.027

**Published:** 2026-05-20

**Authors:** Aslı Cemşitoğlu, Ece Gökyayla, Işıl Karaarslan, Banu Yaman

**Affiliations:** a Department of Dermatology and Venereology, Ege University Faculty of Medicine, Izmir, Turkey; b Department of Pathology, Ege University Faculty of Medicine, Izmir, Turkey

**Keywords:** basal cell carcinoma, cutaneous sarcoma, pleomorphic dermal sarcoma, radiotherapy

## Introduction

Pleomorphic dermal sarcoma (PDS) is a malignant mesenchymal neoplasm of the skin that predominantly affects elderly individuals and typically arises on chronically sun-exposed areas of the head and neck.^1^ It is considered the aggressive end of the atypical fibroxanthoma spectrum, sharing histomorphologic overlap while exhibiting adverse features such as infiltrative growth, deep tissue involvement, tumor necrosis, and an increased risk of local recurrence and distant metastasis.^2^^,^^3^

Although radiotherapy is a well-recognized risk factor for secondary sarcomas arising within previously treated fields, this association has been primarily established for deep soft tissue sarcomas rather than cutaneous tumors.^4^ Accordingly, the relevance of prior radiotherapy to PDS remains insufficiently defined. In particular, PDS developing after radiotherapy poses diagnostic and conceptual challenges, and data regarding latency intervals and long-term behavior in this setting remain limited. Against this background, we describe a patient who developed PDS within a previously irradiated cutaneous field more than 3 decades after definitive radiotherapy for basal cell carcinoma (BCC).

## Case presentation

A 63-year-old male patient applied with a rapidly enlarging lesion on the right side of his nose. His medical history was notable for a BCC diagnosed 33 years earlier in the same anatomical region. At the time of the initial BCC diagnosis, the patient had been evaluated by a multidisciplinary tumor board and, due to concerns regarding potential surgical morbidity related to the tumor location, declined surgical excision and underwent definitive external beam radiotherapy, delivered in a fractionated schedule to a cumulative dose of 45 Gy. According to the patient’s medical history, no local recurrence, new primary skin tumors, or chronic ulceration developed within the irradiated field or elsewhere in the head and neck region during the subsequent follow-up period.

**Fig 1:**
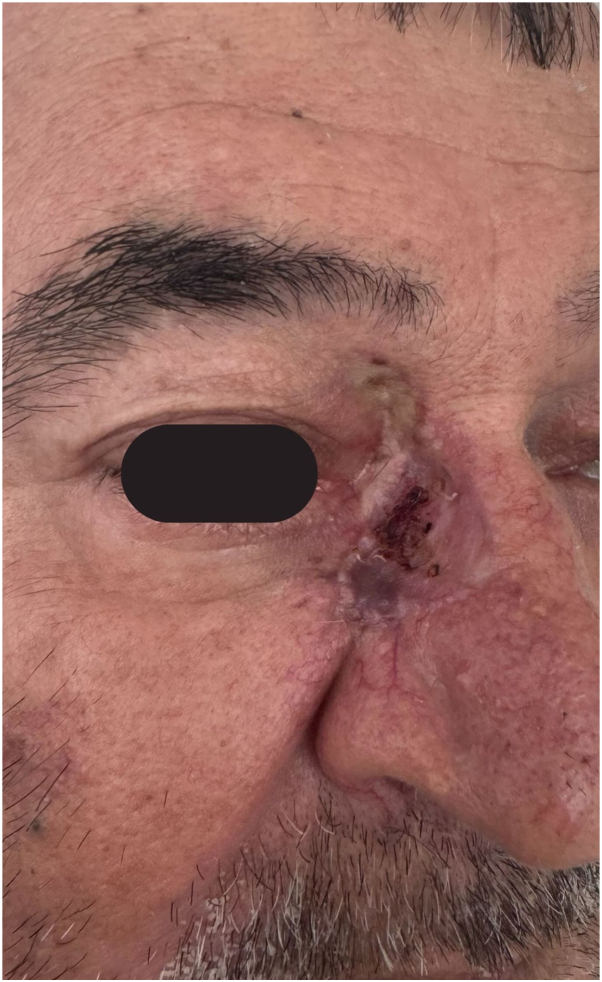
An infiltrative plaque with an irregular surface and focal crusting, extending from the right medial canthus to the nasolabial fold.

**Fig 2:**
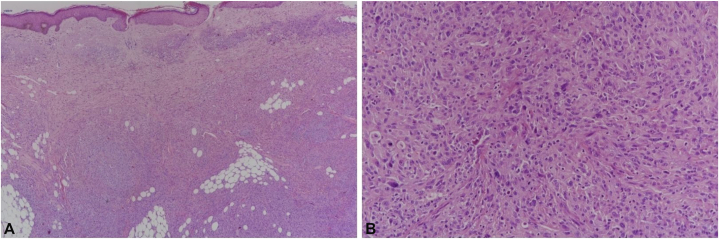
A dermal-based malignant neoplasm composed of pleomorphic spindle cells arranged in an infiltrative growth pattern (Hematoxylin & eosin, **A,** ×40, **B,** ×200 magnification).

On current dermatologic examination, an infiltrative plaque with an irregular surface and focal crusting was observed, extending from the right medial canthus to the nasolabial fold (Fig 1). Given the rapid growth pattern and the clinical suspicion of malignancy arising within a previously irradiated area, a biopsy was performed. Histopathological examination demonstrated a dermal-based malignant neoplasm composed of markedly pleomorphic spindle cells arranged in an infiltrative growth pattern, with invasion into the underlying muscle tissue (Fig 2). The tumor cells showed pronounced nuclear atypia and frequent mitotic figures. Immunohistochemical analysis revealed an elevated Ki-67 proliferation index of approximately 18%. Additional immunohistochemical studies showed negativity for epithelial markers (AE1/AE3, EMA), melanocytic markers (S100, SOX10, HMB45, MITF), vascular markers (CD31, ERG, CD34), and myogenic markers (smooth muscle actin, desmin), thereby excluding epithelial, melanocytic, vascular, and myogenic neoplasms. Among the markers tested, CD10 showed focal positivity. Based on the combined histopathological and immunohistochemical findings, a diagnosis of PDS was established.

## Discussion

In this case, although a definitive causal relationship cannot be established, the strict topographic concordance between the tumor and the previously irradiated cutaneous field supports a possible association with prior radiotherapy.^1^^,^^3^^,^^4^ The prolonged interval of 33 years between radiotherapy and tumor development is noteworthy and lies at the upper end of latency periods reported for radiation-associated sarcomas.^4^ This exceptionally long latency underscores that the oncogenic effects of ionizing radiation on cutaneous mesenchymal tissues may persist for decades, even after prolonged periods of apparent clinical quiescence.^1^, ^2^, ^3^, ^4^

Nevertheless, an alternative explanation deserves consideration. PDS is strongly associated with chronic ultraviolet (UV) exposure and characteristically arises on severely photodamaged skin of the head and neck in elderly individuals.^1^^,^^2^ The prior development of a BCC in the same anatomical site indicates substantial cumulative actinic damage in that field, and multiple primary cutaneous malignancies of different histological types arising within severely photodamaged skin are well recognized. Accordingly, the subsequent occurrence of PDS in the same region may equally represent a second, independent UV-driven neoplasm rather than a true radiation-induced event, and a purely coincidental relationship cannot be excluded on topographic grounds alone.

From a molecular standpoint, PDS typically displays a high tumor mutational burden dominated by a UV-signature pattern of C > T transitions at dipyrimidine sites, with recurrent alterations in TP53, CDKN2A, the TERT promoter, NOTCH1/2, and FAT1.^2^ This molecular profile overlaps substantially with that of cutaneous squamous cell carcinoma and reinforces chronic UV radiation as the principal mutagenic driver of sporadic disease. In contrast, radiation-associated sarcomas generally demonstrate complex karyotypes with TP53 and CDKN2A alterations but typically lack the distinctive UV signature observed in sporadic PDS.^4^ Molecular profiling was not performed in our patient, which represents a limitation of the present report; such analysis could have helped to distinguish between a predominantly UV-driven and a radiation-associated pathogenesis.

This case highlights that the latency period of radiation-associated sarcomas may extend up to 33 years. PDS should be recognized as a potential radiation-associated sarcoma that may arise long after radiotherapy. In the management of indolent cutaneous tumors such as BCC, radiotherapy should be reserved for carefully selected patients, with consideration of possible long-term complications. Finally, patients who have received radiotherapy for any indication require long-term dermatologic follow-up, as sustained surveillance of irradiated fields is essential for the timely detection of secondary malignancies.

### Declaration of generative AI and AI-assisted technologies in the writing process

No generative AI or AI-assisted technologies were used in the preparation of this manuscript.

## Conflicts of interest

None disclosed.

## References

[1] Lo A.C.Q., McDonald S., Wong K.Y. (2021). Case of pleomorphic dermal sarcoma with systematic review of disease characteristics, outcomes and management. BMJ Case Rep CP.

[2] Saleh J.S., Whittington C.P., Bresler S.C., Patel R.M. (2024). Pleomorphic dermal sarcoma. Surg Pathol Clin.

[3] Ørholt M., Abebe K., Rasmussen L.E. (2023). Atypical fibroxanthoma and pleomorphic dermal sarcoma: local recurrence and metastasis in a nationwide population-based cohort of 1118 patients. J Am Acad Dermatol.

[4] Mito J.K., Mitra D., Doyle L.A. (2019). Radiation-associated sarcomas: an update on clinical, histologic, and molecular features. Surg Pathol Clin.

